# Complete genome sequence of *Vibrio anguillarum* strain NB10, a virulent isolate from the Gulf of Bothnia

**DOI:** 10.1186/s40793-015-0060-7

**Published:** 2015-09-02

**Authors:** Kåre Olav Holm, Kristina Nilsson, Erik Hjerde, Nils-Peder Willassen, Debra L. Milton

**Affiliations:** Department of Chemistry, Faculty of Science and Technology, UiT: The Arctic University of Norway, 9037 Tromsø, NO Norway; Department of Molecular Biology, Umeå Centre for Microbial Research, Umeå University, Department of Molecular Biology, 901 87 Umeå, SE Sweden

**Keywords:** *Vibrio anguillarum*, Fish pathogen, Vibriosis, Marine fish, Genome comparisons

## Abstract

*Vibrio anguillarum* causes a fatal hemorrhagic septicemia in marine fish that leads to great economical losses in aquaculture world-wide. *Vibrio anguillarum* strain NB10 serotype O1 is a Gram-negative, motile, curved rod-shaped bacterium, isolated from a diseased fish on the Swedish coast of the Gulf of Bothnia, and is slightly halophilic. Strain NB10 is a virulent isolate that readily colonizes fish skin and intestinal tissues. Here, the features of this bacterium are described and the annotation and analysis of its complete genome sequence is presented. The genome is 4,373,835 bp in size, consists of two circular chromosomes and one plasmid, and contains 3,783 protein-coding genes and 129 RNA genes.

**Electronic supplementary material:**

The online version of this article (doi:10.1186/s40793-015-0060-7) contains supplementary material, which is available to authorized users.

## Introduction

*Vibrio anguillarum* is a marine pathogen that causes a fatal hemorrhagic septicemia, termed vibriosis, in cultured and wild fish as well as in mollusks and crustaceans [1, 2]. *Vibrio anguillarum* is also known under the name *Listonella anguillarum* [3], which is a later heterotypic synonym [4]. Twenty-three serotypes of *V. anguillarum* are reported and of these, only serotypes O1, O2, and to a lesser extent O3, are the main causes of vibriosis in fish [5–7]. Although vaccines and other preventive measures are in use, vibriosis still has a devastating economical impact on the contemporary larviculture and aquaculture industry worldwide [1, 2].

Despite a significant body of research, our understanding of the virulence mechanisms of *V. anguillarum* is far from complete [2, 8]. A recent assessment of 15 serotypes O1, O2, and O3 isolates in a sea bass larvae model indicated that the virulence of *V. anguillarum* is highly complex requiring multiple instead of a few crucial virulence determinants [9]. Whole genome sequencing of different isolates will further our research to elucidate the vital factors this pathogen utilizes to cause disease.

Recently, the complete genome sequences of two *V. anguillarum* serotype O1 strains have been determined. Strain 775 is an isolate from Coho salmon (*Oncorhynchus kisutch*) in the United States Pacific coast and strain M3 was isolated in China from Japanese flounder (*Paralichthys olivaceus*) [10, 11]. In this study, the complete genome sequence of *V. anguillarum* NB10, a virulent, serotype O1 strain, isolated from diseased fish on the Swedish coast of the Gulf of Bothnia, is presented [12].

## Organism information

### Classification and features

**Fig. 1:**
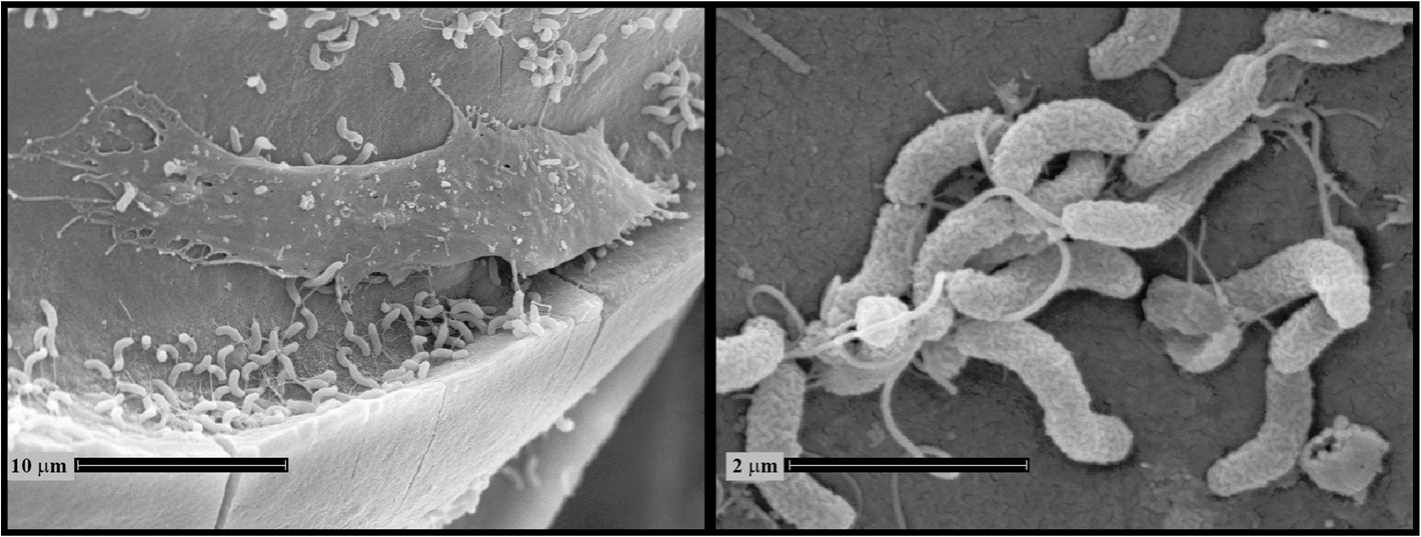
Scanning electron microscopy of *V. anguillarum* NB10 attached to a scale from an infected rainbow trout. The left image was taken at a 3,500× magnification and shows that the bacterium colonizes the groove of a growth ring. A highly motile skin epithelial cell called a keratocyte is shown above the growth ring and *V. anguillarum* evades internalization by the keratocytes [51]. The right image is a higher magnification (20,500×) of *V. anguillarum* cells attached to the surface of the fish scale. In this image, the curved, rod-shape of the bacterium is visible as well as the single polar flagellum. To obtain samples, rainbow trout, 15 g in weight, were infected with *V. anguillarum* NB10 *via* bathing in infected seawater. At 48 h post-infection at 22 °C, scales were removed from lesions that formed on the skin, washed 3× in phosphate buffered saline, and fixed in 2.5 % gluteraldehyde. Electron micrographs were taken using a Cambridge Stereoscan 360 iXP scanning electron microscope at the Electron Microscopy Platform at the Umeå University Core Facility for Electron Microscopy

**Fig. 2:**
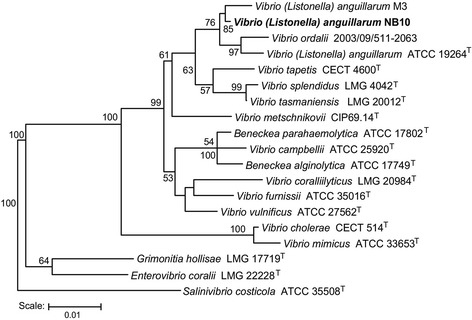
Phylogenetic tree of 16S RNA sequences highlighting the *V. anguillarum* NB10 position relative to other *Vibrio* and *Beneckea* strains. Type strains are indicated with a superscript "T". The strains and their corresponding GenBank accession numbers for 16S rRNA genes are: *V. anguillarum* M3, AY035897; *V. anguillarum* NB10 (chromosome 1, 233,624-235,167 bp), *V. ordalii* 2003/09/511-2063, AY530930; *V. anguillarum* ATCC 19264^T^, X16895; *V. ordalii* ATCC 33509, NR_044851; *V. tapetis* CECT 4600^T^, Y08430; *V. splendidus* LMG 4042^T^, AJ515230; *V. tasmaniensis* LMG 20012^T^, AJ316192; *V. metschnikovii* CIP 69.14^T^, X74711; *B. parahaemolytica* ATCC 17802^T^, AF388386; *V. campbellii* ATCC 25920^T^, X56575; *B. alginolytica* ATCC 17749^T^, X74690; *V. coralliilyticus* LMG 20984^T^, AJ440005; *V. furnissii* ATCC 35016^T^, X76336; *V. vulnificus* ATCC 27562^T^, X76333; *V. cholerae* CECT 514^T^, X76337; and *V. mimicus* ATCC 33653^T^, X74713. For outgroup strains, *Grimonitia hollisae* LMG 17719^T^, AJ514909; *Enterovibrio coralii* LMG 22228^T^, AJ842343; and *Salinivibrio costicola* ATCC 35508T^T^, X74699 were used. The tree uses sequences aligned by the RDP aligner, and uses the Jukes-Cantor corrected distance model to construct a distance matrix based on alignment model positions without the use of alignment inserts, and uses a minimum comparable position of 200. The tree is built with RDP Tree Builder, which uses Weighbor [52] with an alphabet size of 4 and length size of 1000. The building of the tree also involves a bootstrapping process repeated 100 times to generate a majority consensus tree [53]. Bar equals 1% sequence difference. Only significant bootstrap values are indicated

*Vibrio anguillarum* strain NB10 belongs to the class of *Gammaproteobacteria* as part of the *Vibrionaceae* family (Table 1 and Additional file 1: Table S1). The cells display the characteristic curved, rod-shaped morphology of the *Vibrio* genus (Fig. 1) and possess a single polar, sheathed flagellum that is required for colonization of rainbow trout [13–15]. Cells are typically 1-2 microns long and 0.5 microns in width. Colony morphology on tryptone soy agar containing 0.5 % NaCl is a cream-colored, round colony that may sector into translucent and opaque colony types, which may be due to alterations in the expression of outer membrane proteins [16]. The bacterium forms yellow colonies on the vibrio-selective medium thiosulfate-citrate-bile-sucrose agar indicating the fermentation of sucrose. This strain grows at 15-30^ o^C but does not survive at 37^ o^C; in 0.5-4 % NaCl with optimum growth occurring at 1 % NaCl in rich media (unpublished data, D.L. Milton). Strain NB10 is highly virulent for at least two species of fish: rainbow trout (*Oncorhynchus mykiss*) and Atlantic salmon (*Salmo salar*) [13, 17]. Numerous genetically encoded virulence factors have been identified, such as iron transport systems, flagellum/motility, hemolysins, metalloproteases, lipopolysaccharides, exopolysaccharides, repeat toxins, outer membrane proteins, and a type IV pilus [1, 8]. Figure 2 shows the phylogenetic neighborhood of *V. anguillarum* NB10 in a 16S ribosomal RNA based tree.

**Table 1:** Classification and general features of *Vibrio anguillarum* NB10 according to MIGS recommendations [54]

MIGS ID	Property	Term	Evidence code^a^
	Classification	Domain *Bacteria*	TAS [55]
		Phylum *Proteobacteria*	TAS [56]
		Class *Gammaproteobacteria*	TAS [57, 58]
		Order ‘*Vibrionales’*	TAS [56]
		Family *Vibrionaceae*	TAS [59–61]
		Genus *Vibrio*	TAS [59, 60, 62–64]
		Species *Vibrio anguillarum*	TAS [4, 60, 64]
		Strain: NB10	TAS [12, 18]
		Serotype O1	IDA
	Gram stain	Negative	IDA
	Cell shape	Curved rod (vibroid)	TAS [13]
	Motility	Motile	TAS [13]
	Sporulation	Non-sporeforming	IDA
	Temperature range	Mesophile 15-30 °C	IDA
	Optimum temperature	24 °C	IDA
	pH range; Optimum	pH 6 - pH 9; pH 7	NAS
	Carbon source	Highly diverse	NAS
MIGS-6	Habitat	Marine fish	TAS [12, 18]
MIGS-6.3	Salinity	Slightly halophilic, optimum 1 % NaCl	IDA
MIGS-22	Oxygen requirement	Aerobe and facultative anaerobe	IDA
MIGS-15	Biotic relationship	Parasitic	TAS [12, 18]
MIGS-14	Pathogenicity	Pathogen, marine fish	TAS [18]
	Biosafety level	1	NAS
	Isolation	Diseased fish	TAS [12]
MIGS-4	Geographic location	Gulf of Bothnia, Norrbyn, Sweden	TAS [12, 18]
MIGS-5	Sample collection	1986	TAS [12, 18]
MIGS-4.1	Latitude	63° 34' 0'' N	TAS [12, 18]
MIGS-4.2	Longitude	19° 49' 0'' E	TAS [12, 18]
MIGS-4.4	Altitude	3 m	TAS [12, 18]

^a^Evidence codes - *IDA*: Inferred from Direct Assay; *TAS*: Traceable Author Statement (*i.e.,* a direct report exists in the literature); *NAS*: Non-traceable Author Statement (*i.e.,* not directly observed for the living, isolated sample, but based on a generally accepted property for the species, or anecdotal evidence). These evidence codes are from the Gene Ontology project [65]

## Genome sequencing information

### Genome project history

*Vibrio anguillarum* strain NB10 is highly virulent for marine fish and was isolated from the Gulf of Bothnia, a brackish sea off the coast of Norrbyn, Sweden [12, 18]. Genome sequencing was performed by Eurofins MWG GmbH and the Norwegian Sequencing Centre. Finishing and annotation of the genome was performed at the Department of Molecular Biology at Umeå University and at the Department of Chemistry at UiT:The Arctic University of Tromsø, respectively. The genome project has been deposited at the European Nucleotide Archive under the ID number 251627 and accession number PRJEB5701. The accession number for plasmid p67-NB10 is LK021128, for chromosome 1 is LK021130, and for chromosome 2 is LK021129. A summary of the project information is shown in Table 2 and Table S1.

**Table 2:** Project information

MIGS ID	Property	Term
MIGS 31	Finishing quality	Completed
MIGS-28	Libraries used	400-600-bp fragments (454), >10-kb fragments (PacBio)
MIGS 29	Sequencing platforms	Roche 454 Life Sciences
		Pacific Biotechnologies PacBio
MIGS 31.2	Fold coverage	60 ×
MIGS 30	Assemblers	Staden-gap4, Newbler (Roche/454 GS FLX), SMRTanalysis, version 2.0.1, HGAP module (Celera and Quiver)
MIGS 32	Gene calling method	Glimmer3, tRNAscan-SE 1.21, Rfam, RNAmmer
	Locus Tags	VANGcI, VANGcII, VANGp67
	Genbank ID	GCA000786425
	GenBank Date of Release	September 1, 2014
	GOLD ID	Gp0102007
	BIOPROJECT	PRJEB5701
MIGS 13	Source Material Identifier	NB10
	Project relevance	Aquaculture, fish pathogen

### Growth conditions and genomic DNA preparation

*Vibrio anguillarum* NB10 was grown in tryptone soy broth containing 1 % sodium chloride with shaking at 24 ^o^C overnight. For the Roche 454 and PacBio genomic sequencing, genomic DNA was extracted using the Qiagen DNeasy blood and tissue kit according to the manufacturer’s instructions. For gap closures, the genomic DNA, which was used as template for Sanger sequencing, was extracted using the Qiagen Blood and Cell Culture Midi Kit according to the manufacturer’s instructions.

### Genome sequencing and assembly

The genome was sequenced using the Roche/454 GS FLX system equipped with Data Analysis Software Modules v.2.3 [19]. A total of 442,045 reads representing 20-fold coverage of the genome were assembled using the Roche genome assembler Newbler. The assembly resulted in 112 contigs >500 bp. Custom primers were designed to anneal to the ends of the contigs. Gaps between contigs were closed by PCR amplification followed by fragment sequencing. Several gaps could not be closed using this method due to long stretches of repeated sequences. Consequently, the total genome was sequenced a second time using the Pacific Biotechnologies PacBio RS II single-molecule, real-time sequencing technology. Library construction, which contains >10-kb fragments, and sequencing using the PacBio RS II system were performed according to Pacific Biosciences instructions, which may be found on their website [20]. A total of 60,000 reads with a genome coverage of 60-fold were obtained. The sequence reads were assembled using a hierarchical genome-assembly process module from Pacific Biosciences [21]. The HGAP module utilizes the Smrtanalysis, version 2.0.1 to assemble the raw sequence reads and corrects the longest reads utilizing the smaller reads to find a consensus sequence. The corrected reads were then assembled using the Celera Assembler and Quiver softwares resulting in two large contigs associating with chromosome 1 and 2 as well as one small contig representing the plasmid. Error rate of the completed genome sequence using the PacBio RS II system is less than 17 in 50,000 base pairs and the error rate for the Roche/454 GS FLX system is less than 1 in 100,000 base pairs.

### Genome annotation

Coding sequences were predicted using the Glimmer3 program [22]. The numbering of CDSs for each chromosome follows clockwise from the end of the predicted origins of replication at 367 nt and 366 nt for chromosome 1 and 2, respectively. These CDSs were translated, and used to search the National Center for Biotechnology Information nonredundant database as well as the Uniprot and InterPro databases followed by manual curation to assign functional annotation. Using the Basic Local Alignment Search Tool [23], homology searches of all CDSs were done against the Clusters of Orthologous Groups database [24] enabling the assignment of COG functional categories to the CDSs. The tRNAscan-SE 1.21 tool [25, 26] was used to identify tRNA genes; the RNAmmer 1.2 program [27] was used to identify rRNA genes; and the Rfam database [28] and manual curation was used to identify other non-coding RNAs. SignalP server versions 3.0 and 4.0 [29, 30] were used to predict proteins that have signal peptides utilized to target proteins for secretion. The TMHMM server version 2.0 [31] was used to predict transmembrane helices in the proteins. The PHAge Search Tool [32] was used to detect prophage sequences within the genomes. Potential genomic islands were identified using the IslandViewer web server [33] and putative insertion sequences were identified using ISFinder [34]. Putative chromosomal origins of replication were located using the Ori-Finder program [35] followed by manual curation with the help of *V. cholerae* studies that characterized the origins of replication for chromosome 1 [36–38] and chromosome 2 [36, 39, 40] in this organism.

## Genome properties

**Fig. 3:**
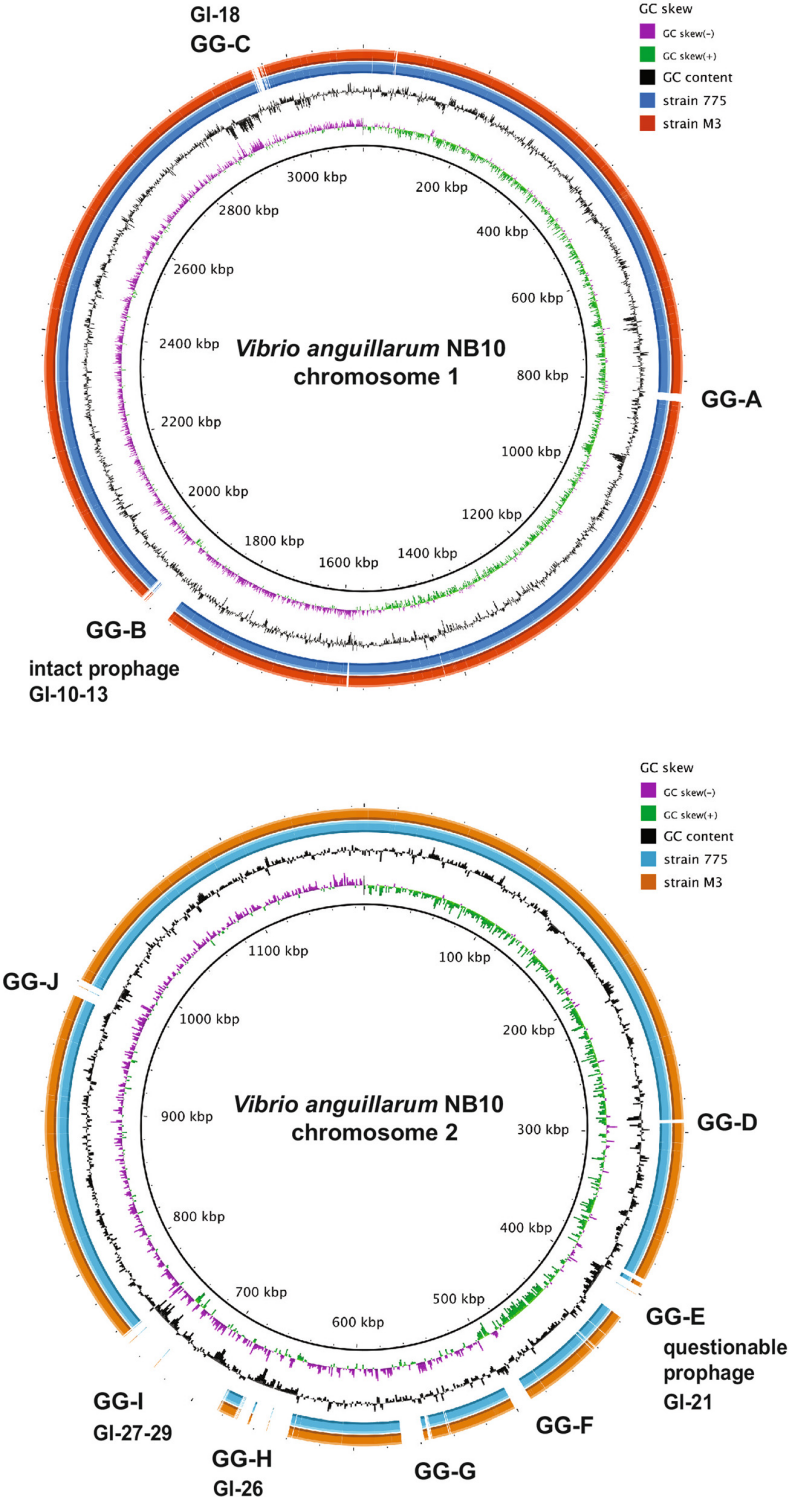
BRIG blast analyses of the two *V. anguillarum* serotype O1 chromosomes. The genome of strain NB10 was used as the reference genome (the inner circle) and compared to the sequenced genomes of strains 775 (blue circle) and M3 (orange circle). Regions showing genomic gaps (GGs) that are missing in strains 775 and M3 are uncolored and are labeled using GG-A to GG-J. In addition, the genomic islands (GIs) and the prophage that are embedded within these genomic gaps are indicated. Refer to Table 7 for exact locations and sizes of the prophages, GIs, and GGs

**Fig. 4:**
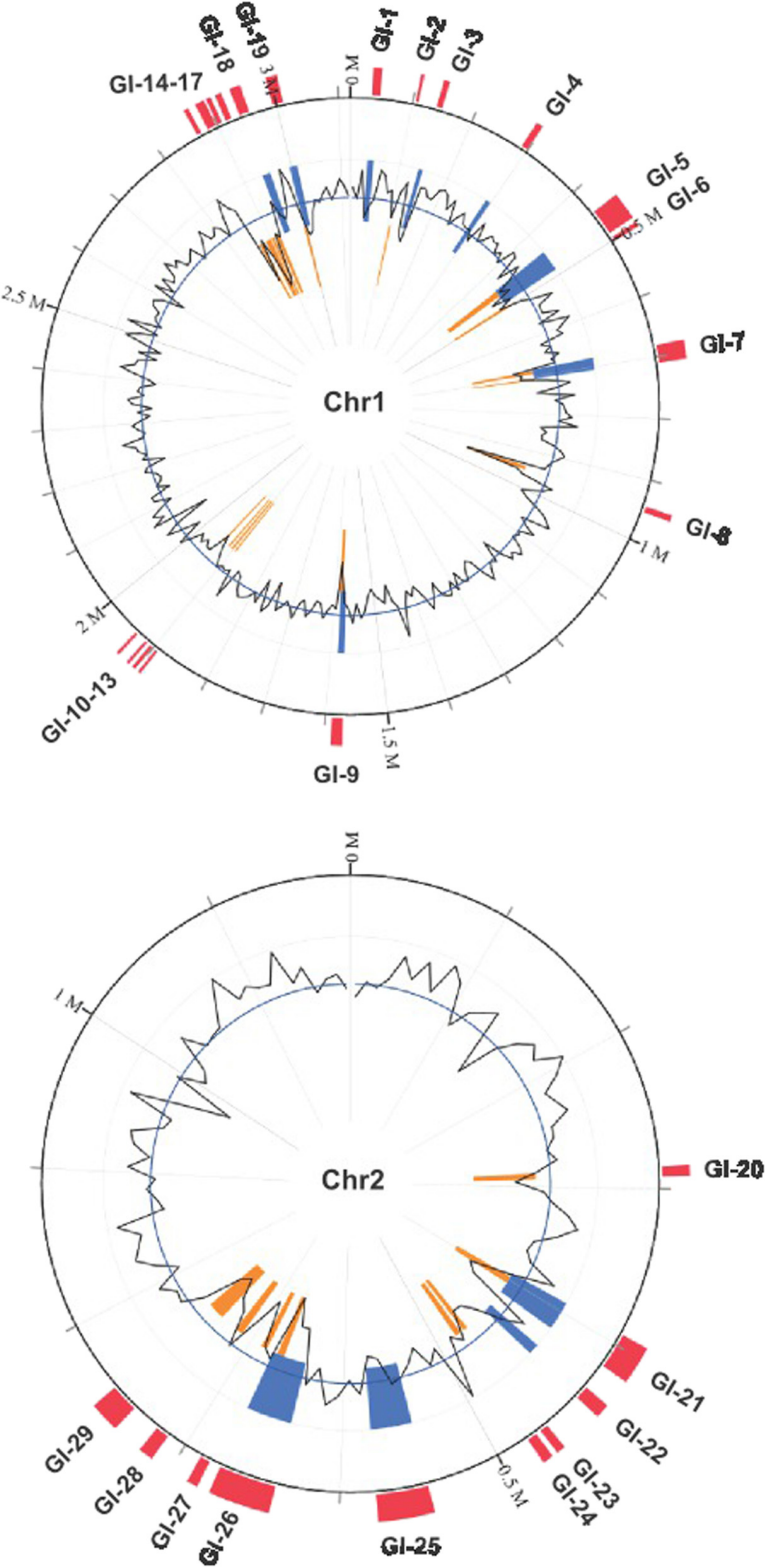
Genomic islands (GIs) of strain NB10 as predicted by the IslandViewer web server. Predicted GIs are colored within the circular image based on the tools used: SIGI-HMM, which predicts GIs based on a hidden Markov model (orange); IslandPath-DIMOB, which predicts GIs based on features associated with genomic islands, such as sequence bias, tRNAs, and integrases and transposases (blue), and an integration of three methods IslandPath-DIMOB, SIGI-HMM and IslandPick, which predicts based on comparative genomics (red). The black line represents the percent GC

The complete genome of *V. anguillarum* NB10 includes two circular chromosomes totaling 4,307,037 bp and one circular plasmid p67-NB10 totaling 66,798 bp, which together give a total genome size of 4,373,835 bp with an average GC content of 44.4 %. Putative *oriC* origins of replication were identified for both chromosomes. For chromosome 1, an *oriCI* region similar to that found in other γ-proteobacteria was found and spans 481 nucleotides (3,119,582 - 367 nt) [36–38]. For chromosome 2, an *oriCII* region similar to that of *V. cholerae* and other *Vibrio* species [36, 39, 40] was found and spans 366 nucleotides (1-366 nt). In addition, an *incII* incompatibility region similar to that of *V. cholerae* was found upstream of the *oriCII* region (1,186,667 - 1,187,342 nt). In *V. cholerae*, *incII* negatively regulates chromosome II replication [36, 39]. Of the 3,912 genes predicted, 3,783 encode proteins, 25 encode rRNAs, 91 encode tRNAs, and at least 13 encode ncRNAs. Fifty-five pseudogenes were found with 33 located on chromosome 1, 21 located on chromosome 2, and 1 located on p67-NB10. Of the predicted CDSs, a functional prediction was made for 84.8 % and 66.9 % were assigned a putative COG function with the remaining annotated as hypothetical proteins. The plasmid is a pJM1-like virulence plasmid that contains 58 protein-coding genes. Four new insertion sequences, named ISVa3, ISVa4, ISVa5, and ISVa6, were identified in this strain and were submitted to ISfinder database [41]. A total of 78 insertion elements were found: 34 on chromosome 1, 31 on chromosome 2, and 13 on p67-NB10. A putative 44.1-kb intact prophage was identified on chromosome 1 and this region is predicted to encode 69 proteins, of which most are phage-related proteins and 33 are hypothetical or uncharacterized proteins. In addition, one questionable prophage was found on chromosome 2 and an incomplete prophage was also found on chromosome 1. The properties and the statistics of the genome are summarized in Tables 3, 4 and 5 and in Figs. 3 and 4.

**Table 3:** Summary of genome: two chromosomes and one plasmid

Label	Size (Mb)	Topology	INSDC identifier	RefSeq ID
Chromosome 1	3.12	Circular	LK021130	NZ_LK021130.1
Chromosome 2	1.19	Circular	LK021129	NZ_LK021129.1
Plasmid p67-NB10^a^	0.07	Circular	LK021128	NZ_LK021128.1

^a^This plasmid is a pJM1-like virulence plasmid [42]

**Table 4:** Genome statistics

Attribute	Value	% of Total
Genome size (bp)	4,373,835	100.0
DNA coding (bp)	3,762,570	86.0
DNA G + C (bp)	1,940,626	44.4
DNA scaffolds	3	
Total genes	3,912	100.0
Protein coding genes	3,783	96.7
RNA genes	129	3.3
Pseudo genes	55	1.4
Genes in internal clusters	447	11.4
Genes with function prediction	3,317	84.8
Genes assigned to COGs	2,620	66.9
Genes with Pfam domains	3,317	84.8
Genes with signal peptides	592	15.1
Genes with transmembrane helices	1,037	26.5
CRISPR repeats	0	0.0

The total is based on either the size of the genome in base pairs or the total number of protein coding genes in the annotated genome

**Table 5:** Number of genes associated with general COG functional categories

Code	Value	% age	Description
J	180	4.8	Translation, ribosomal structure and biogenesis
A	1	0.0	RNA processing and modification
K	219	5.8	Transcription
L	157	4.2	Replication, recombination and repair
B	0	0	Chromatin structure and dynamics
D	34	0.9	Cell cycle control, cell division, chromosome partitioning
V	47	1.2	Defense mechanisms
T	197	5.2	Signal transduction mechanisms
M	161	4.3	Cell wall/membrane biogenesis
N	117	3.1	Cell motility
U	87	2.3	Intracellular trafficking and secretion
O	126	3.3	Posttranslational modification, protein turnover, chaperones
C	170	4.5	Energy production and conversion
G	189	5.0	Carbohydrate transport and metabolism
E	272	7.2	Amino acid transport and metabolism
F	77	2.0	Nucleotide transport and metabolism
H	138	3.6	Coenzyme transport and metabolism
I	78	2.1	Lipid transport and metabolism
P	155	4.1	Inorganic ion transport and metabolism
Q	57	1.5	Secondary metabolites biosynthesis, transport and catabolism
R	332	8.8	General function prediction only
S	234	6.2	Function unknown
-	1163	30.7	Not in COGs

The total is based on the total number of protein coding genes in the genome

## Insights from the genome sequence

### Comparison to other fully sequenced *V. anguillarum* serotype O1 strains

The genome of *V. anguillarum* strain NB10 was compared to the published genomes of *V. anguillarum* serotype O1 strains 775 and M3 [10, 11]. The results are summarized in Table 6. The origin of replication for chromosome 1 *oriCI* is identical in all three strains and for chromosome 2, the NB10 *oriCII* is 99 % identical to that of the 775 and M3. For NB10, we chose to start both chromosomes at their respective origins of replication. The compositional symmetry reflected in the chromosomal GC skews shown in Fig. 3 supports correct trimming of the NB10 replicons. The plasmids in all three strains differ by only a few hundred base pairs, are highly identical with each other, and encode a siderophore-based iron-utilization system that is required for virulence [42, 43]. Compared to strains 775 and M3, the NB10 chromosome 1 is larger by about 56,000 bp and chromosome 2 is increased by about 199,000 bp. To determine if the extra 255,000 bp is unique to the NB10 strain, a BLAST comparison of the three genomes using the BRIG (Blast Ring Image Generator) program [44] and using NB10 as the reference strain was done (Fig. 3). Since horizontal transfer of DNA is a common method for acquiring new DNA in strains, we also screened the three genomes using similar parameters for genomic islands and prophages using the IslandViewer and PHAST search tools and the presence of integrons was determined by identifying *attC* sites, a feature of small mobile gene cassettes that are captured by integrons [45]. These data are summarized in Table 6 and predict that NB10 contains two prophages, a 44.1-kb intact and 45.7-kb questionable, that are not found in strains 775 and M3 and more GIs, of which many are unique, compared to strains 775 and M3. Table 7 presents the location, size, and predicted CDSs for each of the predicted prophages, GIs, and gaps of sequences that are missing in strain 775 and M3. These genomic differences are discussed in detail below.

**Table 6:** Genome comparisons of sequenced *V. anguillarum* serotype O1 strains

Strain	NB10	775	M3
Chromosome 1 (bp)	3,119,695	3,063,912	3,063,587
Chromosome 2 (bp)	1,187,342	988,135	988,134
Plasmid (bp)	66,798	65,009	66,164
Total CDSs^a^	3,782	3,880	3,824
Prophage			
intact	44.1 kb	0	0
incomplete	8.3 kb	8.3 kb	25.7 kb
			8.3 kb
questionable	45.7 kb	0	0
Genomic Islands^b^			
Chr1	19 (208.2 kb)	18 (209.5 kb)	17 (211.6 kb)
Chr2	10 (147.0 kb)	8 (74.3 kb)	5 (62.5 kb)
Number of *attC* sites^c^	65	64	68

^a^Annotation of the NB10 strain included fewer CDSs under 200 bp than that of strains 775 and M3

^b^Numbers represent all genomic islands detected by the IslandViewer tool irrespective of their size or content

^c^Numbers indicate *attC* sites found with a consensus sequence of 5'-TAACAAACGnnTCAAGAGGGAnnGnCAACGC-3'. This sequence makes up a repeat region at the 5’end of the 126-127-bp *attC* sites within the NB10 strain, (unpublished data, K.O. Holm) and indicates the number of integron-associated gene cassettes found in each strain [48]. The *attC* sites occur solely within chromosome 2 but are distributed differently in the three strains

**Table 7:** Unique sequences in *V. anguillarum* NB10 identified by genome comparisons

	Base pairs	Size (kb)	CDSs
Chromosome 1			
*Prophage*			
Intact	1,891,762…1,935,924	44.1	69
Partial	2,964,881…2,973,261	8.3	8
*Genomic Islands*			
GI-1	34,299…47,256	12.9	12
GI-2	104,658…109,015	4.3	5
GI-3	138,956…147,914	8.9	12
GI-4	288,556…298,943	10.3	15
GI-5	445,355…487,284	41.9	37
GI-6	494,899…502,274	7.3	5
GI-7	681,585…709,820	28.2	27
GI-8	942,447…952,116	9.6	9
GI-9	1,572,019…1,589,186	17.1	13
GI-10-13	1,891,012…1,937,302	46.2	61
GI-14-17	2,864,895…2,925,178	60.2	54
GI-18	2,938,796…2,955,240	16.4	17
GI-19	2,994,229…3,011,196	16.9	14
*Genome gaps* ^a^			
GG-A	820,080…832,800	12.7	12
GG-B	1,888,140…1,934,940	46.8	76
GG-C	2,938,440…2,951,880	13.4	13
Chromosome 2			
*Prophage*			
Questionable	401,647…447,433	45.7	22
*Genomic Islands*			
GI-20	286,348…292,555	6.2	5
GI-21	392,736…414,906	22.1	22
GI-22	431,537…439,839	8.3	12
GI-23	464,750…469,836	5.1	5
GI-24	473,125…479,299	6.1	10
GI-25	546,066…578,220	32.1	35
GI-26	639,559…674,888	35.3	24
GI-27	682,155…688,431	6.2	6
GI-28	711,800…720,165	8.3	6
GI-29	738,270…755,649	17.3	10
*Genome gaps* ^a^			
GG-D	292,140…294,360	1.9	3
GG-E	396,120…417,780	21.6	13
GG-F	489,780…500,880	11.1	19
GG-G	557,580…571,320	13.7	11
GG-H	643,380…673,560	34.0	22
GG-I	682,320…756,540	74.2	53
GG-J	971,280…981,740	10.5	9

^a^Gaps indicate regions of the NB10 genome that are not found in the genomes of strains 775 and M3 as detected by BLAST searches using the BRIG program [44]

### Prophage regions

Prophages are bacteriophages that are integrated into the genome and that are diverse mobilizable elements that play a role in horizontal gene transfer. Three putative prophage regions were identified (Table 7 and Fig. 3). Chromosome 1 contains a partial 8.7-kb prophage (Phage_Bacill_G) that is also found in the 775 and M3 genomes and an intact prophage (Phage_Pseudo_vB_PaeS_PMG1) with a size of 44.1 kb that is unique to strain NB10 and that encodes 43 phage-related and 33 hypothetical proteins. Chromosome 2 contains a questionable 45.7-kb prophage (Phage_Stx2) that is also unique to strain NB10. In comparison, strain 775 contains no additional prophages; while, strain M3 contains a second partial 27.5-kb prophage (Phage_Entero_M13) on chromosome 2.

### Genomic Islands (GIs)

Genomic islands (GIs) are clusters of genes, typically >8 kb in size, that likely originate from horizontal gene transfers and that often play a role in the adaptation of bacteria to their environment or host [46]. GIs impact bacterial evolution significantly and the identification of GIs within genomes provides insight into differences between bacterial species and strains. For strain NB10, 29 putative GIs that range from 4.2 kb to 41.9 kb were detected using the IslandViewer tool (Table 7). Of these, 19 are localized to chromosome 1 and 10 to chromosome 2 (Fig. 4). Twenty GIs contain genes that are often associated with the islands, such as tRNAs, transposases, integrases, and phage-related genes. Of the nine that do not contain these typifying genes, GI-4 contains CDSs encoding ribosomal proteins, while GIs-8,14-17 contain CDSs involved in O-antigen biosynthesis. Nineteen of the GIs are found in strains 775 and M3; while, 10 GIs are unique to strain NB10 and their genomic locations are shown in Fig. 3. Overall analyses of the CDSs within all GIs indicate that most encode hypothetical proteins. No obvious virulence genes were detected; however, 7 GIs carried CDSs for toxin-antitoxin systems, in particular that of *hipAB*, which plays a role in antibiotic tolerance and persistence [47].

### Integrons

Integrons are genetic units that contain and disseminate small mobile elements called gene cassettes and thus contribute to genomic diversity [45]. Gene cassettes carry a gene, any gene, and an *attC* site, which is recognized by an integron enabling it to splice cassettes into its integration site. The integration of gene cassettes may occur over and over creating a string of gene cassettes. A consensus *V. anguillarum* NB10 *attC* site sequence repeat was predicted (5'-TAACAAACGnnTCAAGAGGGAnnGnCAACGC-3', unpublished data, K.O. Holm) [48] and used to identify integrons by localizing *attC* sites in the three different genomes. In all strains, *attC* sites were localized only in chromosome 2 and the number of *attC* sites did not differ much. However, in NB10, the location of the *attC* sites were all found within a 154.4-kb region, which represents 3.5 % of the genome and lends support to this location representing a putative superintegron with highly diverse gene cassettes of mostly unknown functions. In particular, this putative superintegron is highly similar to that characterized in the *V. cholerae* strain N16961 since the NB10 integrase shares identity with the *V. cholerae* VchIntIA integrase [48]. In contrast, for strains 775 and M3, the *attC* sites were found at 5 different locations distributed throughout chromosome 2. Whether this difference reflects putative geographical and/or different ancestral characteristics needs further investigation.

### Genomic regions unique to strain NB10

The NB10 genome is around 255,000 bp larger than the genomes of strains 775 and M3. A comparative analysis using BRIG was performed to identify CDSs unique to NB10. Figure 3 indicates the location of 10 genomic gaps ranging from 1.9 kb to 74.2 kb in strains 775 and M3 compared to strain NB10. Table 7 gives the base pair coordinates for each gap. All but one genomic gap (GG-G), which was found on chromosome 1 of strains 775 and M3, contained CDSs unique to strain NB10. The majority of these CDSs encode hypothetical proteins. However, a few CDSs were identified that may provide strain NB10 an advantage either in its host or in its aquatic environment. The GG-A contains a CDS for a haem peroxidase that may play a role in oxidative stress aiding colonization of the host [49]. Numerous CDSs encode putative toxin-antitoxin systems, which have been reported to play a role in antibiotic tolerance, persistence, stress response, and virulence in some bacteria [50]. Many of the CDSs within these genomic gaps may be genes with not yet known functions, as they were not found within other bacterial species using a BLAST search.

## Conclusions

In this study, the complete genome sequence of the *V. anguillarum* strain NB10 serotype O1, a virulent isolate from the Gulf of Bothnia, Norrbyn, Sweden, is presented. Genome comparisons were done with the complete genomes of two other virulent, O1 serotype *V. anguillarum* strains, M3 and 775. Although the genomes of M3 and 775 strains are quite similar in size, the genome from the NB10 strain was shown to contain an extra 255,000 bp that are unique to this strain. The extra DNA is predicted to contain two putative prophages as well as a number of GIs and genomic gaps, all of which are predicted to encode mostly hypothetical proteins with no obvious roles in virulence. The roles of the extra genomic sequences found in the *V. anguillarum* strain NB10 compared to strains 775 and M3 remain to be determined. However, a few genes were found in the extra DNA regions that may be predicted to aid the survival of strain NB10 in its host or its natural habitat, the brackish Baltic Sea, which contains both salty and fresh water [12]. Thus, in comparison to the M3 and 775 strains, which were isolated from different types of geographical regions [10, 11], it is tempting to speculate that the CDSs within the extra sequences may play a role in the ecology of strain NB10.

## Additional file

Additional file 1: Table S1. Associated MIGS record. (DOC 70 kb)

## Abbreviations

BRIG: Blast ring image generator
GG: Genomic gaps
GI: Genomic islands
HAMAP: High-quality automated and manual annotation of proteins
HGAP: Hierarchical genome-assembly process
IS: Insertion elements
Pfam: Protein families
PHAST: Phage search tool
PROSITE: Protein domains families and functional sites
TMHMM: Tied mixture hidden markov model
