# Supplementary material for: Complete genome sequence of Vibrio anguillarum strain NB10, a virulent isolate from the Gulf of Bothnia
Source: Stand Genomic Sci. 2015 Sep 2;10:60. doi: 10.1186/s40793-015-0060-7 (PMC4572688; doi:10.1186/s40793-015-0060-7)
Supplement: Additional file 1: Table S1. — Associated MIGS record. (DOC 70 kb) [file 40793_2015_60_MOESM1_ESM.doc]

***Table S1.*** *Associated MIGS record*

| **MIGS-ID** | field name | Description |
| --- | --- | --- |
| **MIGS-1** | Submit to INSDC/Trace archives | not reported |
| **1.1** | PID |  |
| **1.2** | Trace Archive |  |
| **MIGS-2** | MIGS CHECK LIST TYPE | not reported |
| **MIGS-3** | Project Name | PRJN251627 |
| **MIGS-4** | Geographic Location | Gulf of Bothnia, Norrbyn, Sweden |
| **4.1** | Latitude | 63.56^o^ N |
| **4.2** | Longitude | 19.83^o^ E |
| **4.3** | Depth | not reported |
| **4.4** | Altitude | Marine |
| **MIGS-5** | Time of Sample collection | 1986 |
| **MIGS-6** | Habitat (EnvO) | marine fish |
| **6.1** | temperature | mesophilic 15-30^o^C |
| **6.2** | pH | not reported |
| **6.3** | salinity | slightly halophilic, optimum 1% NaCl |
| **6.4** | chlorophyll | not reported |
| **6.5** | conductivity | not reported |
| **6.6** | light intensity | not reported |
| **6.7** | dissolved organic carbon (DOC) | not reported |
| **6.8** | current | not reported |
| **6.9** | atmospheric data | not reported |
| **6.10** | density | not reported |
| **6.11** | alkalinity | not reported |
| **6.12** | dissolved oxygen | not reported |
| **6.13** | particulate organic carbon (POC) | not reported |
| **6.14** | phosphate | not reported |
| **6.15** | nitrate | not reported |
| **6.16** | sulfates | not reported |
| **6.17** | sulfides | not reported |
| **6.18** | primary production | not reported |
| **MIGS-7** | Subspecific genetic lineage | Serotype O1 |
| **MIGS-9** | Number of replicons | 3 |
| **MIGS-10** | Extrachromosomal elements | 1 |
| **MIGS-11** | Estimated Size | 4,373,835 |
| **MIGS-12** | Reference for biomaterial or Genome report | not reported |
| **MIGS-13** | Source material identifiers | not reported |
| **MIGS-14** | Known Pathogenicity | virulent |
| **MIGS-15** | Biotic Relationship | Parasitic |
| **MIGS-16** | Specific Host | marine fish |
| **MIGS-17** | Host specificity or range (taxid) | >50 species |
| **MIGS-18** | Health status of Host | not reported |
| **MIGS-19** | Trophic Level | not reported |
| **MIGS-22** | Relationship to Oxygen | aerobic / facultative anaerobic |
| **MIGS-23** | Isolation and Growth conditions | isolated from diseased fish, growth in tryptone soy broth with 1% NaCl at 15-30^o^C |
| **MIGS-27** | Nucleic acid preparation | Qiagen DNeasy blood and tissue kit |
| **MIGS-28** | Library construction | 400-600-bp fragments (Roche/454 GS FLX)  >10-kb fragments (PacBio RSII) |
| **28.1** | Library size | not reported |
| **28.2** | Number of reads | 60,000 |
| **28.3** | vector | not reported |
| **MIGS-29** | Sequencing method | Roche 454 Life Sciences  Pacific Biotechnologies PacBio |
| **MIGS-30** | Assembly |  |
| **30.1** | Assembly method | Staden-gap4  Newbler (Roche/454 GS FLX)  SMRTanalysis, version 2.0.1  HGAP module (Celera and Quiver) |
| **30.2** | estimated error rate | Roche/454 GS FLX: <1 in 100,000 bp  PacBio RS II: <17 in 50,000 bp |
| **30.3** | method of calculation | not reported |
| **MIGS-31** | Finishing strategy | Complete |
| **31.1** | Status | Complete |
| **31.2** | coverage | 60 x |
| **31.3** | contigs | 300 (Roche 454 Life Sciences)  3 (PacBio system) |
| **MIGS-32** | Relevant SOPs | not reported |
| **MIGS-33** | Relevant e-resources | not reported |
